# Punish and Voice: Punishment Enhances Cooperation when Combined with Norm-Signalling

**DOI:** 10.1371/journal.pone.0064941

**Published:** 2013-06-12

**Authors:** Giulia Andrighetto, Jordi Brandts, Rosaria Conte, Jordi Sabater-Mir, Hector Solaz, Daniel Villatoro

**Affiliations:** 1 Institute of Cognitive Science and Technology, National Research Council (CNR), Rome, Italy; 2 European University Institute, Florence, Italy; 3 Universitat Autònoma de Barcelona, Institut d’Anàlisi Econòmica, Consejo Superior de Investigaciones Científicas (CSIC), and Barcelona Graduate School of Economics, Barcelona, Spain; 4 Institut d’Investigació en Intelligència Artificial, CSIC, Barcelona, Spain; 5 Nuffield College, University of Oxford, Oxford, United Kingdom; Universidad Carlos III de Madrid, Spain

## Abstract

Material punishment has been suggested to play a key role in sustaining human cooperation. Experimental findings, however, show that inflicting mere material costs does not always increase cooperation and may even have detrimental effects. Indeed, ethnographic evidence suggests that the most typical punishing strategies in human ecologies (e.g., gossip, derision, blame and criticism) naturally combine normative information with material punishment. Using laboratory experiments with humans, we show that the interaction of norm communication and material punishment leads to higher and more stable cooperation at a lower cost for the group than when used separately. In this work, we argue and provide experimental evidence that successful human cooperation is the outcome of the interaction between instrumental decision-making and the norm psychology humans are provided with. Norm psychology is a cognitive machinery to detect and reason upon norms that is characterized by a salience mechanism devoted to track how much a norm is prominent within a group. We test our hypothesis both in the laboratory and with an agent-based model. The agent-based model incorporates fundamental aspects of norm psychology absent from previous work. The combination of these methods allows us to provide an explanation for the proximate mechanisms behind the observed cooperative behaviour. The consistency between the two sources of data supports our hypothesis that cooperation is a product of norm psychology solicited by norm-signalling and coercive devices.

## Introduction

In natural social contexts, individuals often use punishment to discourage the violation of norms [1]–[2], i.e., prescribed behaviours shared and enforced by a community [3]–[5]. As a consequence, punishment is typically considered one of the primary means for social control and the transmission of norms. However, in behavioural experiments, and in particular in public goods games, punishment is usually modelled only as the imposition of material costs [6]–[8] and the transmission of norms is restricted by design (but see [9]–[14]). Subjects can infer how to behave only from the punishment received. In such experimental settings, material punishment may not only be ineffective in sustaining norm compliance, but may even lead to the erosion of the gains obtained from increased cooperation [6]–[7], [15]–[18].

Our hypothesis is that when material punishment is combined with norm communication, subjects cooperate more and less punishment is needed. We propose that successful human cooperation results from the interaction of the norm psychology [3], [19]–[20] and the cognitive machinery for instrumental decision-making. Norms inform individuals about how they are prescribed to behave. Material punishment makes the expected consequences of violating them more certain thus making norms salient in subjects’ mind [4], [21]–[23]. Salient norms lead, ceteris paribus, to higher compliance [23].

## Experiment

### 1. Experiments with Human Subjects

To test our hypothesis, we build an experimental design utilizing a standard public-goods game with costly punishment [6]. Our novel treatment is one in which the transmission of what is the norm can be achieved by combining peer communication and material punishment to form what we call a sanction. In two control conditions sanctions are decoupled into material punishment and communication; these two treatments are called punishment and message respectively. This design allows us to investigate the relative and combined effect of norm communication and material punishment in promoting cooperation.

Our experiment is built as follows. In all treatments, twelve fixed groups of four subjects interact over thirty rounds. The first (1–10) and the last (21–30) ten rounds are identical across treatments. At every round, each member *i* of a group independently chooses an integer contribution level, C_i_, between 0 and 20, with the following resulting payoff:
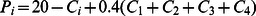



After each round all the members of the group are informed about the contribution levels of each of the other three members. Since players’ identities persist, subjects can trace one another’s behaviour throughout the rounds. In rounds 11–20, treatments differ.

In the punishment treatment, after each round, participants can assign an integer amount between 0 and 10 punishment units to each of the other group members. Each assigned punishment unit costs the punished member 3 units and the punishing member 1 unit. Each punished group member is informed about the ID of all punishers at the end of each round.

In the message treatment, after each round, participants cannot inflict material punishment but can send the following message to the other group members, choosing between options 1), 2) and 3): “One should contribute X (indicating the demanded token amount), because 1) in this way we are all better off; 2) it is what one should do, and 3) if not it will have consequences for you”. Options 1) to 3) capture three different reasons for contributing: 1) achievement of a joint benefit; 2) a sense of duty and 3) a purely individualistic motivation. Sending the message entails no material cost for the sender or for the receiver (details are provided in Text S1, see Figures S1, S2, S3, S4).

In the sanction treatment, participants have, after each round, the opportunity to both assign punishment points and send a normative message (details are provided in Text S1).

A treatment similar to our sanction treatment has been used in [10], [14]. In [10], Noussair and Tucker study experimentally the joint effects of what they call formal and informal sanctions. Formal sanctions consist in assigning material punishment points like in our set-up. However, their informal sanctions are very different from ours. They consist in the possibility of assigning disapproval points to others without any material consequence (see also [11]). These informal sanctions are a way of giving a negative rating, more or less disapproval, to other group members’ decisions. The authors find that being able to use both types of sanctions leads to a higher increase in cooperation than their separate use. However, differently from our findings, they show that the combined use of informal and formal sanctions does not prevent a detrimental effect of punishment on group’s earnings. They explain their results in terms of “a wider array of sanctions, which provides a greater ability to nuance the disciplinary action taken against free riders.” In contrast to the negative ratings used in [10], in our design the message has a positive normative content, by containing a quantitative prescription of how much to contribute together with a reason for the prescription. We consider this a more direct and incisive way of conveying information about norms.

Recently, in [14], Janssen et al. present an experimental environment in which costly punishment can be combined with communication to study social-ecological systems. In their set-up participants can communicate extensively and decide whether or not to adopt a punishment system and how much the fines should be. They also allow for the different temporal orders of the availability of punishment, communication and both and their results depend to some extent on the order of the treatments. Among other results, they find that communication with punishment does not lead to as long-lasting cooperative behavior as communication without punishment, a result at odds with ours. In contrast with [14], in our work we chose to keep the treatments completely separate and to restrict the communication possibilities to the sending of normative messages and to make the punishment system exogenously present or absent. In [25], [26] another way of conveying normative information is explored. It is shown how symbolic punishment, consisting in showing an unhappy face, has an effect on cooperation.

Apart from the differences in the experimental design, the novelty of our work consists in combining experiments with humans and agent-based simulations in order to provide an explanation for the proximate mechanisms behind human cooperation. Currently, the need for cross-methodological research is increasingly felt among the social scientists for the sake of both empirical validation and modeling. While laboratory data show us the impact of manipulated independent variables on human behavior, agent based modeling helps us investigate the internal mechanisms, which generate such behavior.

## Results and Discussion

### 2. Experiments with Human Subjects: Results and Discussion

**Figure 1:**
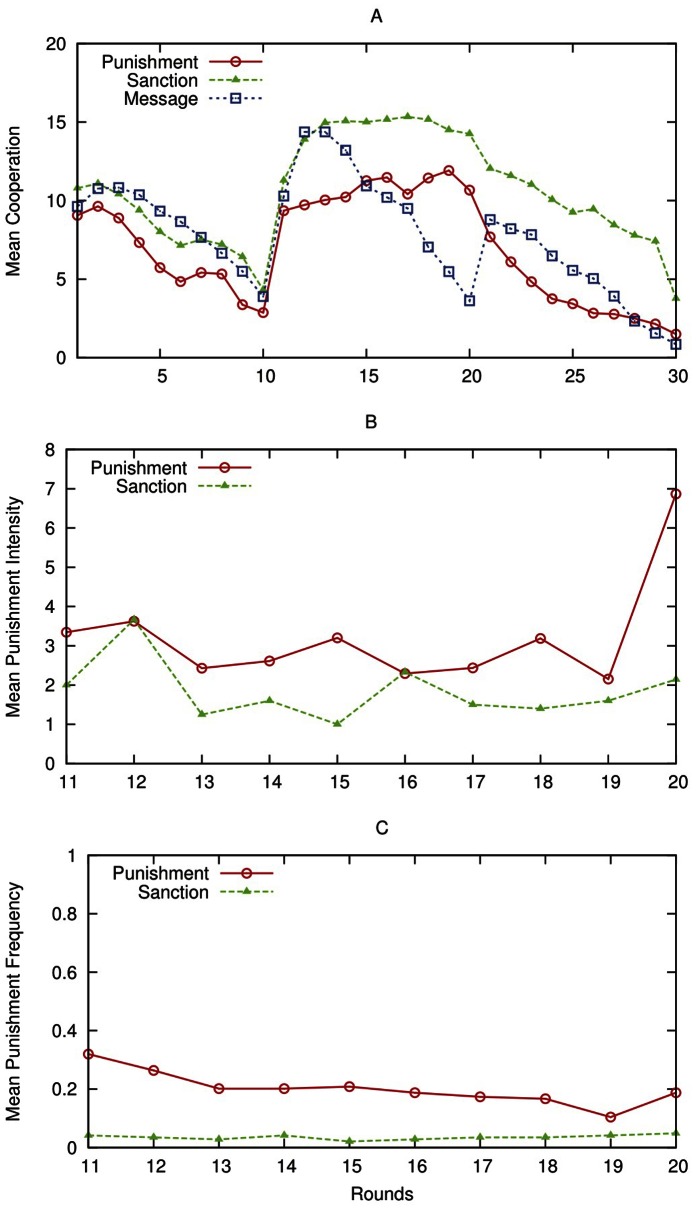
(A–C) Results of the Experiments with Human Subjects. Panel A depicts the contribution levels obtained in the human experiments. Panel B depicts the punishment intensity observed in the human experiments. Mean punishment intensity is defined as the average number of punishment units sent, whenever punishment is used, i.e. all instances of zero punishment are excluded. Panel C depicts the punishment frequency observed in the human experiments. Punishment frequency measures the average number of times punishment is used, regardless of the number of punishment units sent.

Figure 1 A–C shows average contribution levels in the three treatments, average punishment intensity in the punishment and sanction treatments, and average punishment frequency in the same two treatments. During rounds 1–10 contribution levels decline in all treatments, with average contributions being 8.33, 6.25 and 8.23 in the message, the punishment and the sanction treatment respectively; the Kruskal-Wallis test does not find any differences between the three data-sets (p = 0.1821). For rounds 16–20, average contribution levels contribution levels are 9.90, 10.65 and 14.46 in the message, the punishment and the sanction treatment respectively. This implies that contributions in the sanction treatment are significantly higher by 36% than in the punishment treatment and significantly higher by 52% than in the message treatment; in this case the Kruskall-Wallis test finds significant differences between the three treatments (p = 0.07). In the last ten rounds, when punishment and normative message opportunities are switched off, contributions decay in all three cases to average levels of 5.05, 3.75 and 9.08 in the message, the punishment and the sanction treatment respectively. Now cooperation levels in the sanction treatment exceed by 142% those obtained in the punishment treatment and by 79% those in the message treatment, with the Kruskal-Wallis test finding a significant difference (p = 0.04). Overall, the contribution levels are quite low with respect to those reported in [6], [16], where the same parameter values are used. An experiment comparing contribution behaviour in student populations in Spain, the Netherlands, the US and Japan (see [27]) finds that contributions in Spain (Pompeu Fabra University) are the lowest, although the difference is not statistically different.

Using the Mann-Whitney test, the difference between average contributions in the sanction and the punishment treatments in rounds 11–20 is significant (p = .048). There is no significant difference in contributions between sanction and message for rounds 11–15 (p = .7290), but contributions are significantly higher in sanction than in message for rounds 16–20 (p = .0179). Average contributions are significantly higher in the message than in the punishment treatment (p = .0833) in rounds 11–15, but no difference in rounds 16–20 (p = .2987).

The average number of punishment points sent is 1.73 times higher in the punishment than in the sanction treatment (see also Table S1 and Figure S5 included in the Supporting Information). Using the Mann-Whitney test, we find that average punishment points allocated per member is significantly higher in the punishment than in the sanction treatment (p = .0005) (1.24167 vs. 0.1625 average points sent). Moreover, in the punishment treatment, the frequency of punishment is 5.68 times higher than in the sanction treatment. Using the Mann-Whitney test, we find that in the punishment treatment, the frequency of punishment is significantly higher than in the sanction treatment (p = .0004). Figure S4 in the Supporting Information shows mean punishment as a function of the punished subject’s contribution minus that of the punisher for the two treatments involving punishment. Table S3 in the Supporting Information provides support for the idea that those players who contribute less than asked to are strongly punished.

Due to higher contributions and lower punishment, average net earnings are 31% higher in the sanction than in the punishment treatment for rounds 11–20 and 16% higher than in the message treatment for rounds 16–20. Unlike in the other two treatments, earnings in the sanction treatment are 12.38% higher in rounds 11–20 than in rounds 1–10 (see also [14], [24]). By using sanction, the gains from higher contributions are not offset by the associated punishment costs. Payoffs are higher in the sanction than in the punishment treatment for rounds 11–15 (p = .0010) and for rounds 16–20 (p = .0055). Payoff levels are not higher in the message than in the sanction treatments for rounds 11–15 (p = .8174), but are significantly higher in sanction than in message for rounds 16–20 (p = .0242). Payoffs are significantly higher in the message than in the punishment treatment (p = .0007) in rounds 11–15, but no difference in rounds 16–20 (p = .4529).

We can use the within-subjects nature of our design to compare contribution levels in the second block with those of the first block. A Wilcoxon test finds that, unlike in the other two treatments, in the sanction treatment payoffs are significantly higher in rounds 11–20 than in rounds 1–10 (p = .0096) and than in rounds 21–30 (p = .0022). This is in contrast to the results reported in [6]–[7], [18].

In both the message and sanction treatments, messages from peers help subjects soon to identify the prescribed amount of contribution and to form expectations about the consequences of violations. In both treatments, subjects’ expectations and their behaviours rapidly converge. Figures S1 and S2 in the Supporting Information show that the percentages of individuals that sent a message and the average required contribution levels in rounds 11–20 are quite similar for the two relevant treatments. Table S2 in the Supporting Information shows that in both treatments subjects that ask for high contributions are those who contribute at high levels. Figure S3 in the Supporting Information shows that the use of the three different messages differs somewhat across the two relevant treatments. Specifically, behaviour in the sanction treatment exhibits a stronger concentration on message 1 (“In this way we are all better off”), while in the message treatment message frequencies are a little more dispersed.

The value added by material punishment to norm communication consists in strengthening the normative expectations, thus increasing the norm salience in subjects’ minds. As shown by the results in the message treatment (and differently from [14]), when norms are verbally transmitted but not enforced by material punishment compliance soon declines. Since a high number of participants deviates from the contribution due, the norm becomes less salient and inefficient in sustaining compliance. In the sanction treatment, subjects immediately meet the prescription and the possible use of punishment only sustains the contribution level reached. In contrast, in the punishment treatment, in which information about norms can only be inferred from the material cost received, the cooperation level reached is substantially lower and the costs for achieving it is higher than in the sanction treatment. When modelled only in material terms, punishment is scarcely effective in helping subjects to find out the norm. Far from coordinating, subjects separately preceed by trial and error.

## Experiment

### 3. Simulation Experiment

To test our hypothesis about the decision process underlying observed behaviour, we develop an agent-based model that explicitly incorporates the norm psychology as part of its decision making. The motivation for norm compliance is modelled as dependent of both the salience of the norm and the instrumental decision-making (see Text S1 for details). The agent-based model is a dynamic one in which the propensity to follow the norm changes over time depending on the behaviour observed during the interaction and, in this sense, goes beyond the purely static social preference models.

Simulations reproduce the public-good game used in the laboratory and the three experimental conditions: punishment, message and sanction. The goal of the simulation is to check that the result obtained by the agents in the simulation resembles that of the humans in the laboratory, supporting therefore that the explanation given in terms of the norm psychology is a plausible explanation for the observed human behaviour.

Depending on the relevant treatment, agents can, like humans, decide whether or not to cooperate, punish, and send messages. Cooperation choices are binary: each agent chooses whether to cooperate (C) by contributing a fixed amount or to defect by contributing nothing (D). Cooperation choices depend on a probability that varies at each round as a function of the force of both an individual drive and a normative drive (see Text S1 for details).

The individual drive (ID) approximates the instrumental decision-making processes. It pushes agents to maximize their own personal utility regardless of what the norm prescribes and is updated according to a winner-stay-losers-change algorithm [28]. The more an action increases the agent’s payoffs, the higher the probability it will be chosen. The individual drive directs the choice toward cooperation (C) only when the benefit of defecting is lower than the benefit of cooperating. Agents’ payoffs depend on their actions, and they are lowered according to the costs sustained when imposing punishment or sanction and when receiving them.The normative drive (ND) models the motivation to comply as dependent on norm salience. Norm salience is a parameter updated by each agent at every round according to the information gathered by observing the behaviour of the other agents and by communicating with them (see Table S4 included in the Supporting Information for details). The values of this parameter have been calibrated on the data extracted from [23].

The cooperation probability changes over time depending on the values that the individual drive and the normative drive of each agent take. The cooperation probability varies across agents, thus generating heterogeneity withing the population. The tendency to cooperate is always positively affected by the normative drive and possibly by the individual drive, if cooperation returns higher payoffs than defection. In this case the two drives complement each other. Conversely, it will be negatively affected by the individual drive, when defection returns higher payoffs than cooperation. In this second case, one drive goes against the other (see Text S1 for details about the Agents’ Strategies Updating).

The probability of punishing is negatively affected by the number of defectors, while the probability of sending a message indicating that the norm prescribes to cooperate (C) (associated or not with punishment) is a direct function of the perceived salience of the norm (see Text S1 for details). Punishment is costly and its intensity is binary (high or low). The cost of being punished is always greater than the net cost of cooperating. Figure S6 in the Supporting Information shows the use of high and low punishment and of normative messages in the simulations for each of the three treatments.

## Results and Discussion

### 4. Simulation Experiment: Results and Discussion

**Figure 2:**
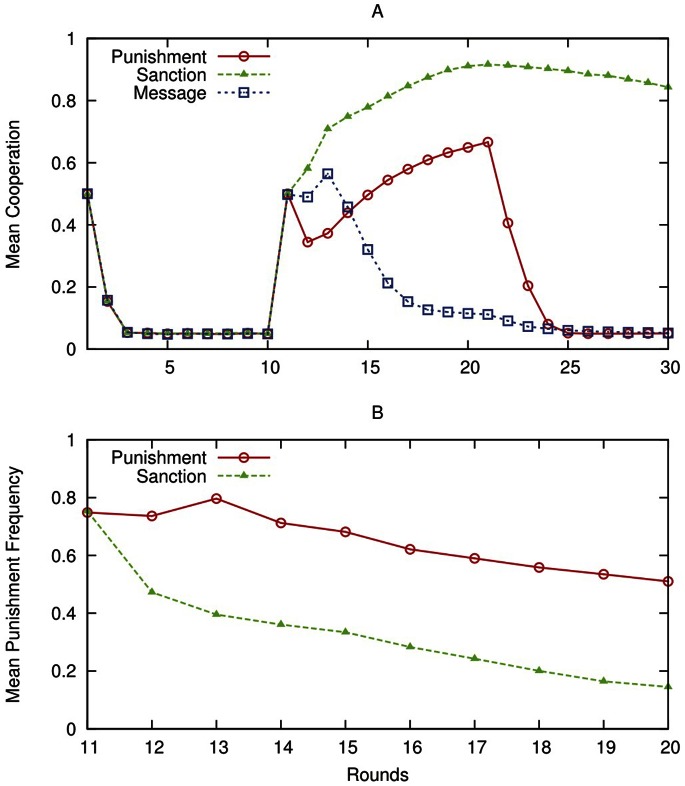
(A–B) Results of the simulation experiments. Panel A depicts the cooperation levels observed in the simulation experiments. Agents are initialized with Individual Weight = 0.5; Normative Weight = 0.5; Initial Punishment Probability = 0.5; Forgetting Probability = 0.3 (for a parameter space exploration, see Text S1). The simulation experiment generates trends similar to the ones obtained with human subjects (compare Figure 1A and Figure 2B). After round 10, cooperation levels are higher in the sanction treatment than in the punishment treatment, because of the combined effect of the normative message and the monetary punishment. Panel B depicts the punishment frequency in the simulation experiments. Simulation results show that the frequency of punishment is significantly higher in the punishment treatment than in the sanction treatment, resulting in a less violent society even obtaining a higher cooperation rates as shown in the previous figure (compare Figure 1C and Figure 2B).

Figure 2A–B shows simulation results about cooperation levels and punishment frequency. The simulation data are consistent with the human data (confront Figure 1A–C and Figure 2A–B; Figures S8, S9, S10 show alternative parameterizations of the simulation model).

In the sanction treatment, from round 11 to 30, agents reach higher and more stable contribution levels than in the punishment treatment, and punish less. Hence, the strength of the normative drive is higher in the sanction than in the punishment treatment. In the latter, the normative drive is poorly solicited because of the lack of explicit information about the norm, thus the behaviour is mainly guided by the individual drive (details are presented in Figure S5 and a discussion of these results is provided in Text S1). In the message treatment the initial contribution level is similar to the sanction treatment, but later declines substantially. As norm violations are not followed by material punishment, the salience of the norm C rapidly decreases, and the individual drive turns agents toward D. After round 20, when agents cannot punish or send messages anymore, the level of cooperation remains higher in the sanction than in the other two treatments, since agents have learned the norm and the normative drive strengthens the resilience of cooperative behaviour (Figure S7 shows average payoffs of the agents in the three treatments). In the third block (rounds 21–30), the decline of cooperation level in the agent-based simulations is faster than in the human data, and also faster than in [29]. One possible explanation is that – once a high level of cooperation is reached – human behaviour is somewhat less adaptive than that of the simulated agents. When humans realize in round 21 that, given the absence of punishment and messages, cooperation tends to fall they may try to actively resist the decline in cooperation. This effort is eventually futile, but does slow down the contribution decline to some extent.

## Conclusions

Both our results show that norm communication boosts cooperation and material punishment serves to maintain it. This implies that it is not punishment that prescribes people how to behave, but norms. Punishment is a supplementary mechanism through which norms are made salient and the expected consequences of violating them more certain. Norms create an environment in which the infliction of punishment has to be less frequent and severe and its detrimental effect is mitigated [1]–[2], [30]. This may help to understand the evolution of punishment [31].

Consistency between computational and laboratory data supports our inferences on the proximate mechanisms that promote human cooperation. Humans are provided with a norm psychology to detect norms and track their salience. In real world environments, which typically involve the combined use of norms and coercive devices, human cooperation results from the interplay of norm psychology and instrumental decision-making.

## Materials and Methods

### 5. Ethics Statement

Our experiment is about decision-making and involves no physical intervention. All our experimental sessions were conducted with the informed consent of all adult participants, who knew that they were free to withdraw from participation at any time. Individuals invited to one of our sessions had previously voluntarily registered in the LINEEX laboratory of the University of Valencia database. To do that they had to go to LINEEX website. On that website the rules of the lab were available. Informed consent was indicated by electronic acceptance of an invitation to attend an experimental session. The voluntary registration in the electronic database documents participants’ acceptance. The experiments were conducted following the procedures established by LINEEX laboratory of the University of Valencia. Our study was approved by the Director of the LINEEX laboratory (Professor Enrique Fatás) at an ethics review and project proposal meeting that is required for all experiments conducted at the LINEEX facilities.

### 6. Experiments with Human Subjects

#### Participants and procedure

The laboratory experiments with humans were conducted between March and May 2011 at the LINEEX laboratory of the University of Valencia. 144 participants were recruited from a pool of undergraduate students from the University of Valencia and voluntarily participated in the 3 sessions of our experiment. Special care was exerted to recruit students from many different disciplines to increase the likelihood that the subjects had never met before. Each participant was allowed to take part in only one session. On arrival, participants were immediately led to separate cubicles. Instructions on general behavior in the lab and specific instruction about the game to be played were read by a mother tongue laboratory assistant. The experiment was programmed by using the z-tree platform [32].

#### Instructions (for the sanction treatment)

The purpose of this experiment is to study how individuals make decisions in certain contexts. The instructions are simple and if you follow them carefully you will be paid a cash amount of money privately, since nobody will know about the earnings of the other participants. You can ask questions at any moment by raising your hand. Apart from these questions, any type of communication between you is not allowed and may lead to exclusion from the experiment.

1. For your participation in this experiment you obtain an initial payment of 200 ECU (Experimental Currency Unit). The experiment has 30 rounds. In each round you are part of the same group of 4 participants, the composition of which is determined in round 1 and does not vary during the whole experiment.
2. The groups are formed by the participants in this room, and the groups will be randomly formed by the server at the beginning of the experiment.
3. Within each group, each participant will randomly receive an identification number at the beginning of the experiment. This number will be used to identify the decisions made by each participant within a group, but nobody will know the identity of the members of the group and all actions that you will take during the experiment will be absolutely anonymous.
4. In each round you will make decisions in two phases. At the end of each phase, you will receive information about the decisions of all the members of your group.
5. The first phase consists in deciding how much to contribute to a common good. At the beginning of the phase you will receive 20 ECUs and you will have to decide how much to invest in the common good and how much to keep for you. Your decision and those of the other participants will affect the payoff you will receive in this phase:

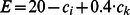
where E is your payoff, *c_i_* is your own contribution and *c_k_* is the total of contributions by the rest of participants
  After deciding how much to contribute to the common good, you have to press the button “continue.” Once you will have pressed it your decision will be final.
6. Once all the participants in your group will have made their decisions, you will on the screen the total amount of ECUs contributed to the common good by each of the members of your group (including your own contribution). This screen will also show how many ECUs you have obtained, calculated using the formula shown above.
7. In the second phase you will be able to assign between 0 and 10 punishment points that will receive the payoffs obtained by the members of the group to which you assign such points. Each punishment point has a cost of 1 ECU for you and an effect of 3 ECUs for the receiver of the punishment. That is, if you assign 1 punishment point to another participant, he/she will have his payoff reduced by 3 ECUs.
  Therefore, you have to decide how many punishment points to assign to each of the member of your group. Once the points are assigned, you have to press the button “continue” and your decision will be final.
  Your payoff will be affected by the assigned points in the following way:
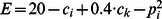
where *p_i_* is the total amount of points that you have assigned to the other participants.
  In addition, in this phase you have the possibility to indicate to each of the other participants what the right behavior is, by completing the following sentence and marking one of the following options.
*One should contribute X, because:*
    - *In this way we are all better off*
    - *It is what one should do*
    - *If not, it will have consequences for you.*
  This message has no direct effect on your payoffs or the payoffs of the receiver of the message.
8. Once all participants in your group will have made their decisions, you will see on the screen how many punishment points have been assigned to you as well as each of the messages that you have received.
  These points have an effect on your final payoff in the rounds, that is calculated in the following way:
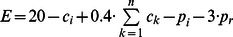

  Where *p_r_* is the total amount of punishment points received from the other participants.
9. Observe that your final payoff can be negative if the cost of your decisions in the second phase is higher than the payoff obtained in the first phase. Note that in any case you can avoid losses through your decisions.
10. At the end of the experiment you will be paid in cash and privately your accumulated payoffs for the whole experiment at the exchange rate of 40ECUs = €1

## Supporting Information

Figure S1 **Percentage of individuals that sent a message over rounds 11–20 in the Experiments with Human Subjects.**
(TIF)

Figure S2 **Average required contribution in tokens over rounds 11–20 in the Experiments with Human Subjects.**
(TIF)

Figure S3 **Percentages of the three verbal messages sent in the message and sanction treatment over rounds 11–20 in the Experiments with Human Subjects.**
(TIF)

Figure S4 **Punishment intensity in the Experiments with Human Subjects depending on punished subject’s contribution minus that of punisher.**
(TIF)

Figure S5 **Dynamics of the Individual and Normative Drives in the Agent Based Model.**
(TIF)

Figure S6 **Amount of punishments, sanctions and messages sent in the Agent Based simulation.**
(TIF)

Figure S7 **Average Payoffs along the Agent Based simulation.**
(TIF)

Figure S8 **Mean Cooperation along the simulation experiment contrasted with the value of the Initial Punishment Probability.**
(TIF)

Figure S9 **Mean Cooperation along the simulation experiment contrasted with the value of the Forgetting Probability.**
(TIF)

Figure S10 **Mean Cooperation along the simulation experiment contrasted with the value of the Individual Weight.**
(TIF)

Table S1 **Determinants of punishment levels in the punishment and sanction treatments in the Experiments with Human Subjects: random-effects tobit regressions.**
(TIFF)

Table S2 **Determinants of suggested contributions in the message and sanction treatments in the Experiments with Human Subjects: random-effects tobit regressions.**
(TIFF)

Table S3 **Determinants of punishment levels in the sanction treatment in the Experiments with Human Subjects: random-effects tobit regressions.**
(TIFF)

Table S4 **Norm Salience Mechanism in the Agent Based Model: Cues and Weights.**
(TIFF)

Text S1 **Supporting Information Document.**
(DOC)
