# Supplementary figures and images for: Punish and Voice: Punishment Enhances Cooperation when Combined with Norm-Signalling
Source: PLoS One. 2013 Jun 12;8(6):e64941. doi: 10.1371/journal.pone.0064941 (PMC3680389; doi:10.1371/journal.pone.0064941)

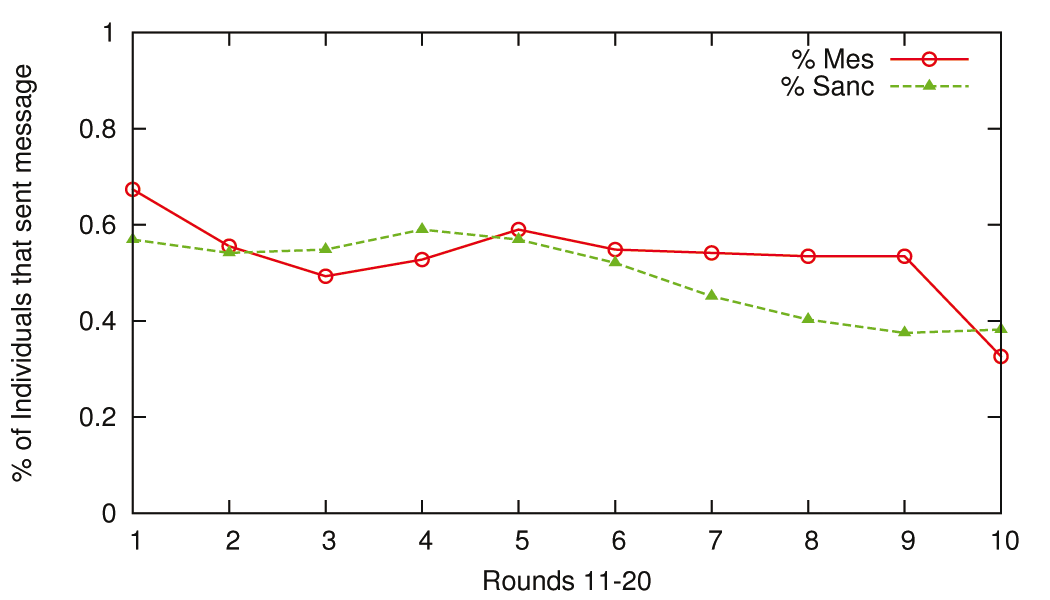

Supplement: Figure S1 — Percentage of individuals that sent a message over rounds 11–20 in the Experiments with Human Subjects. (TIF) [file pone.0064941.s001.tif]

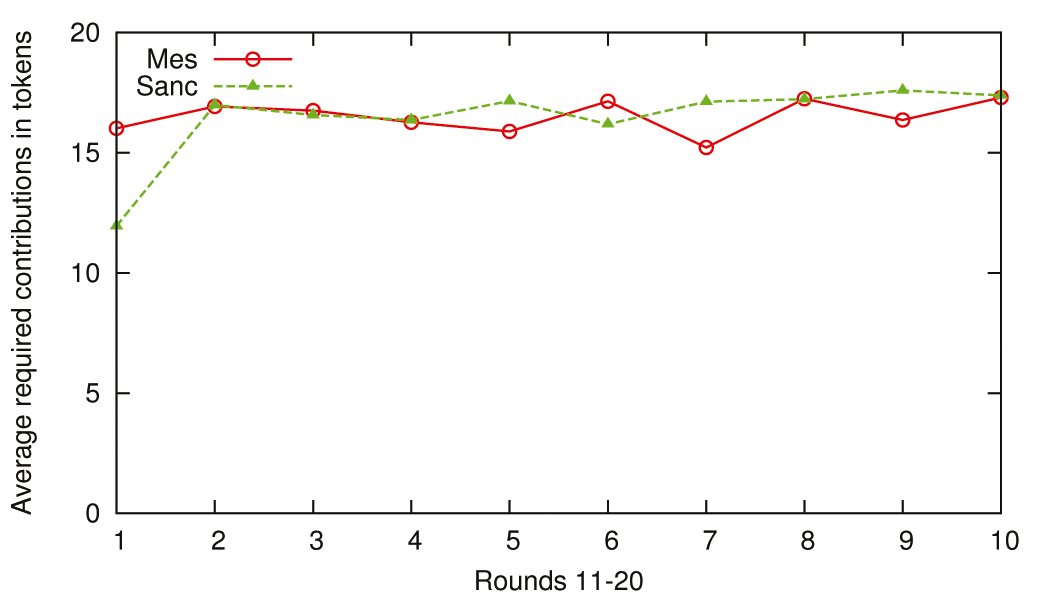

Supplement: Figure S2 — Average required contribution in tokens over rounds 11–20 in the Experiments with Human Subjects. (TIF) [file pone.0064941.s002.tif]

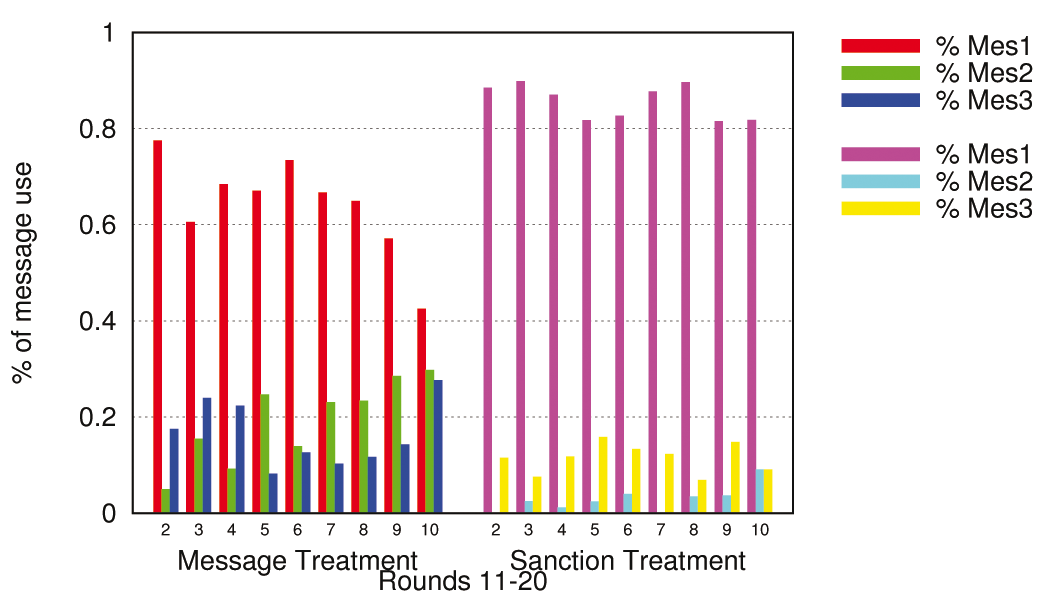

Supplement: Figure S3 — Percentages of the three verbal messages sent in the message and sanction treatment over rounds 11–20 in the Experiments with Human Subjects. (TIF) [file pone.0064941.s003.tif]

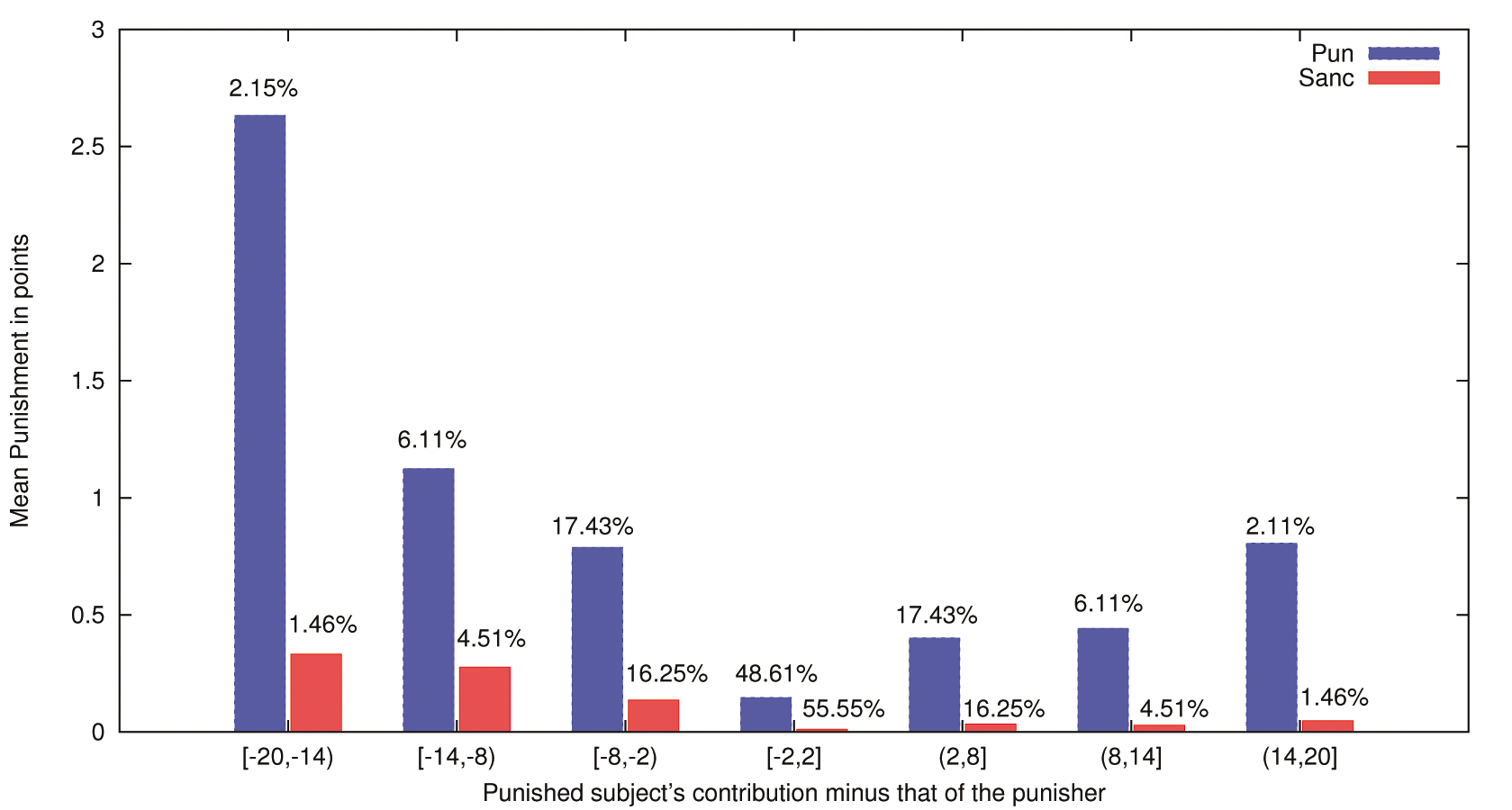

Supplement: Figure S4 — Punishment intensity in the Experiments with Human Subjects depending on punished subject’s contribution minus that of punisher. (TIF) [file pone.0064941.s004.tif]

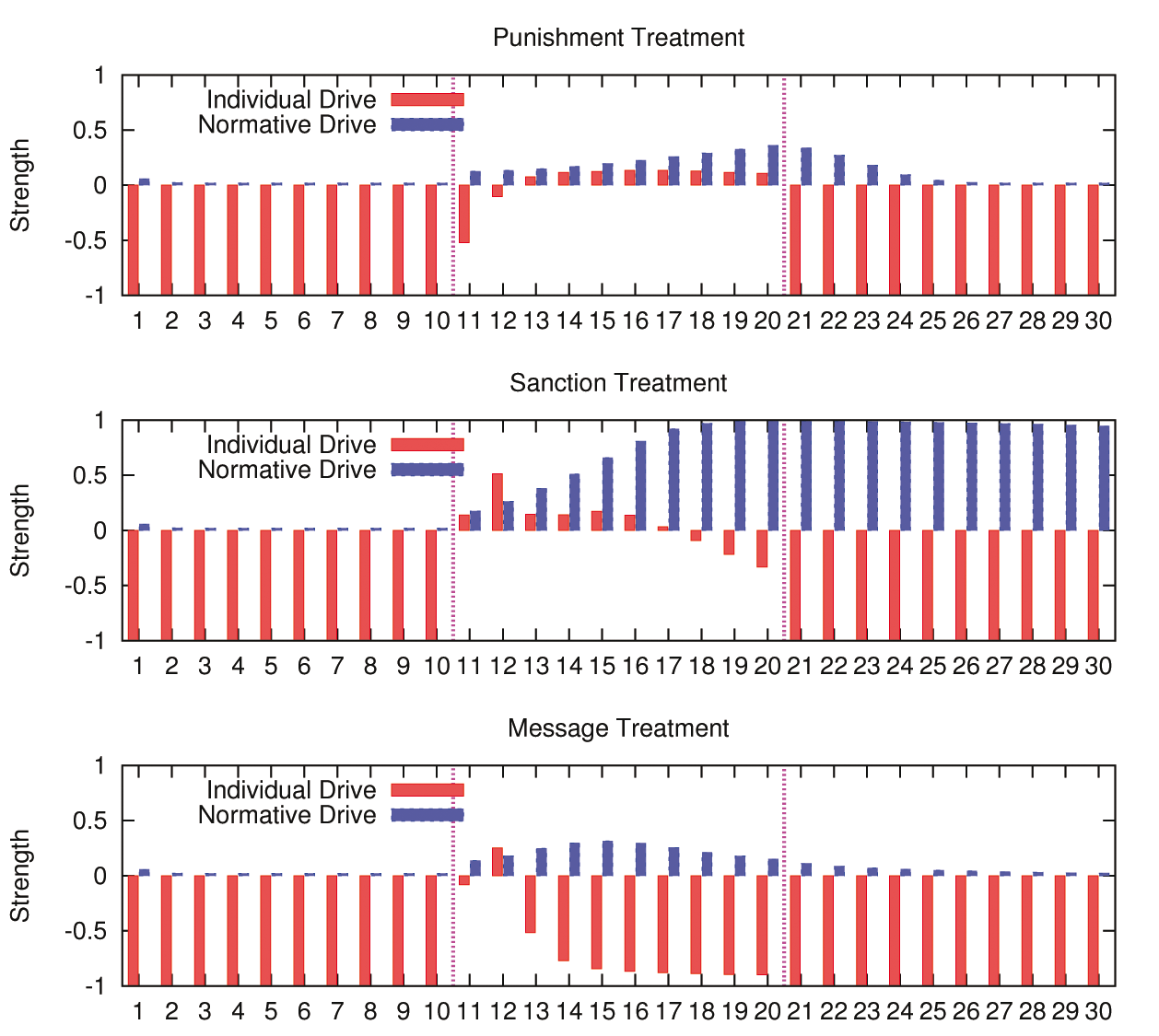

Supplement: Figure S5 — Dynamics of the Individual and Normative Drives in the Agent Based Model. (TIF) [file pone.0064941.s005.tif]

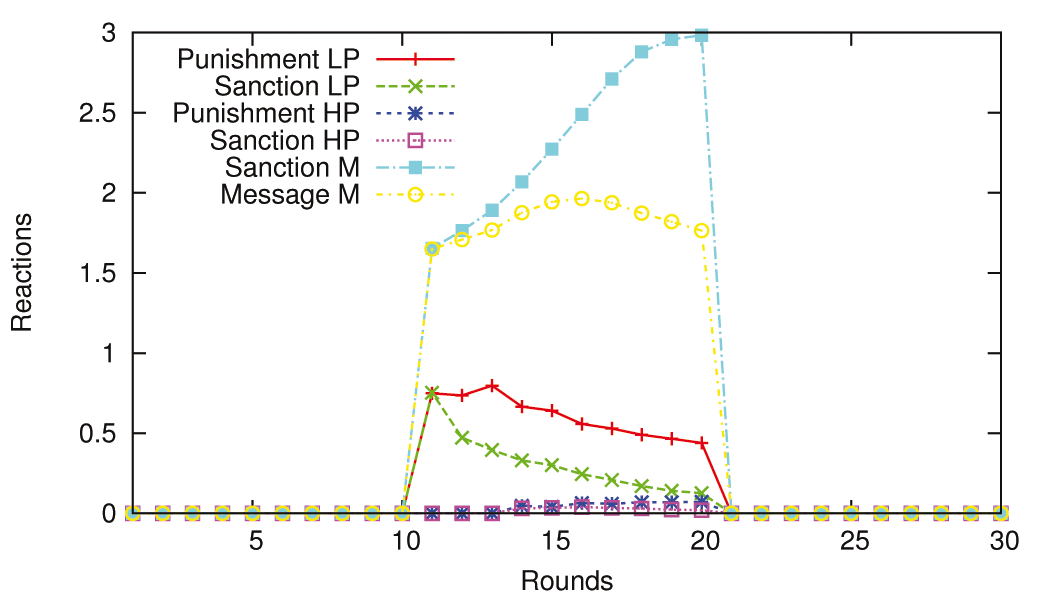

Supplement: Figure S6 — Amount of punishments, sanctions and messages sent in the Agent Based simulation. (TIF) [file pone.0064941.s006.tif]

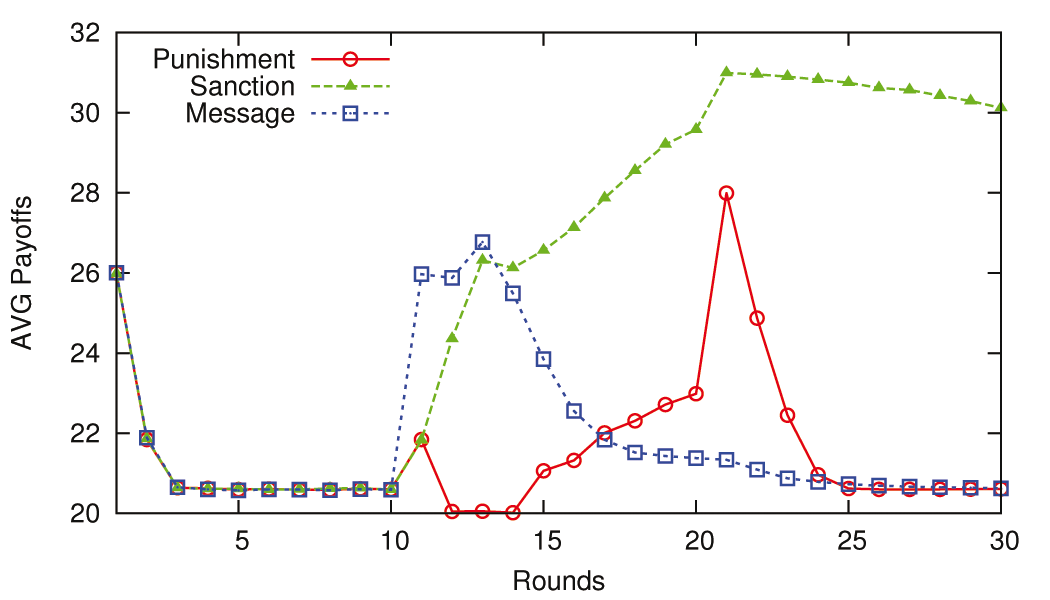

Supplement: Figure S7 — Average Payoffs along the Agent Based simulation. (TIF) [file pone.0064941.s007.tif]

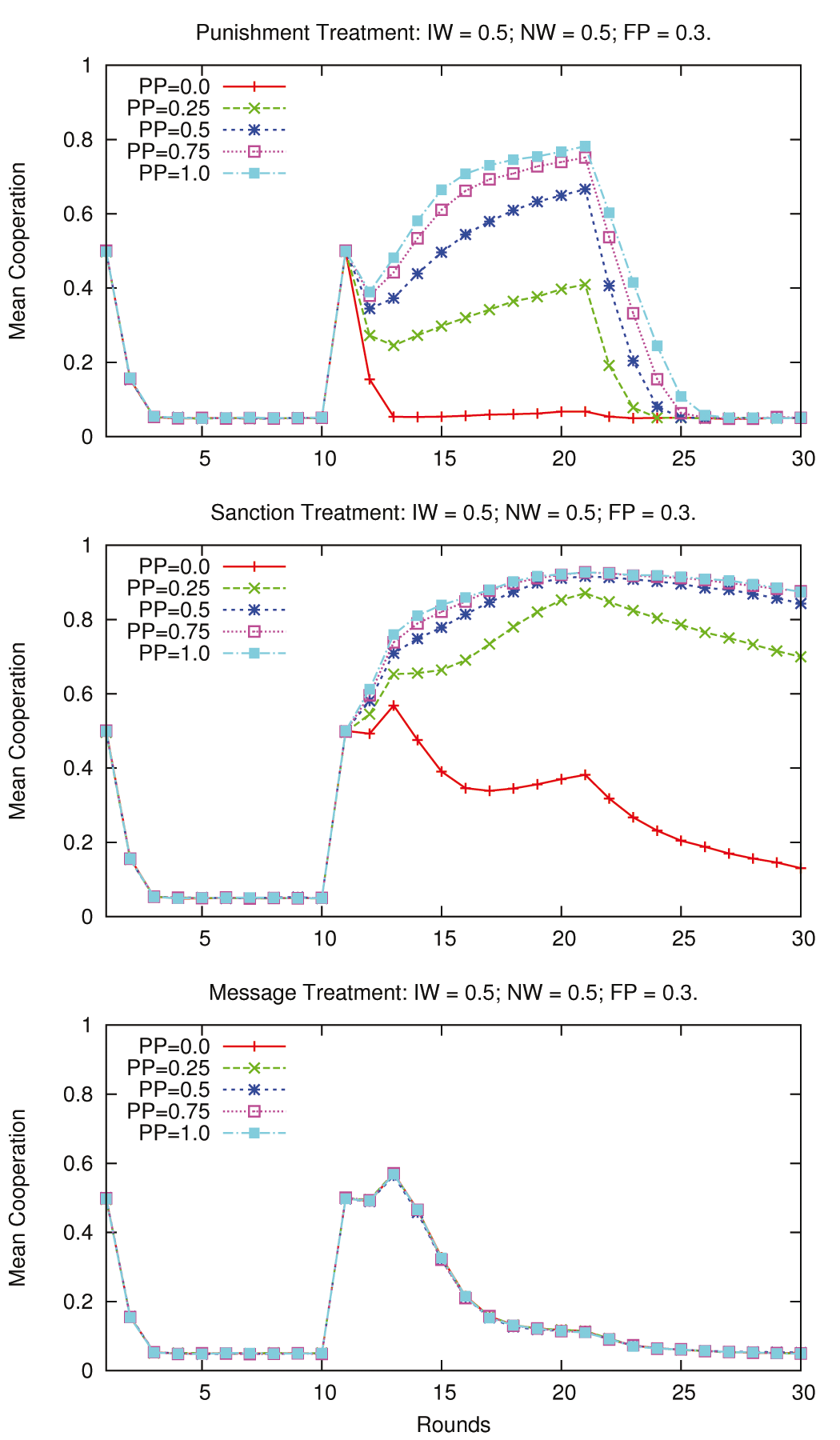

Supplement: Figure S8 — Mean Cooperation along the simulation experiment contrasted with the value of the Initial Punishment Probability. (TIF) [file pone.0064941.s008.tif]

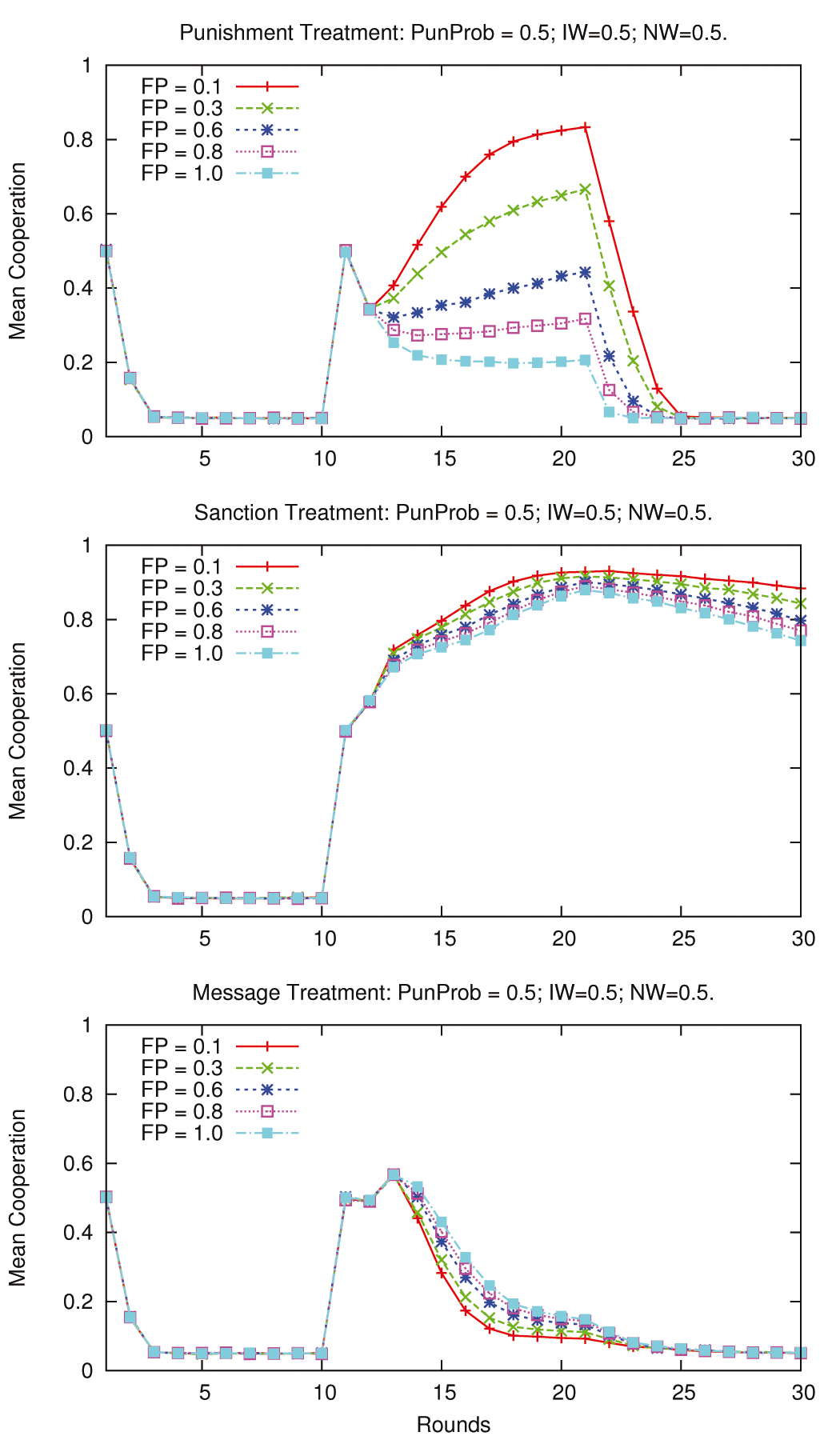

Supplement: Figure S9 — Mean Cooperation along the simulation experiment contrasted with the value of the Forgetting Probability. (TIF) [file pone.0064941.s009.tif]

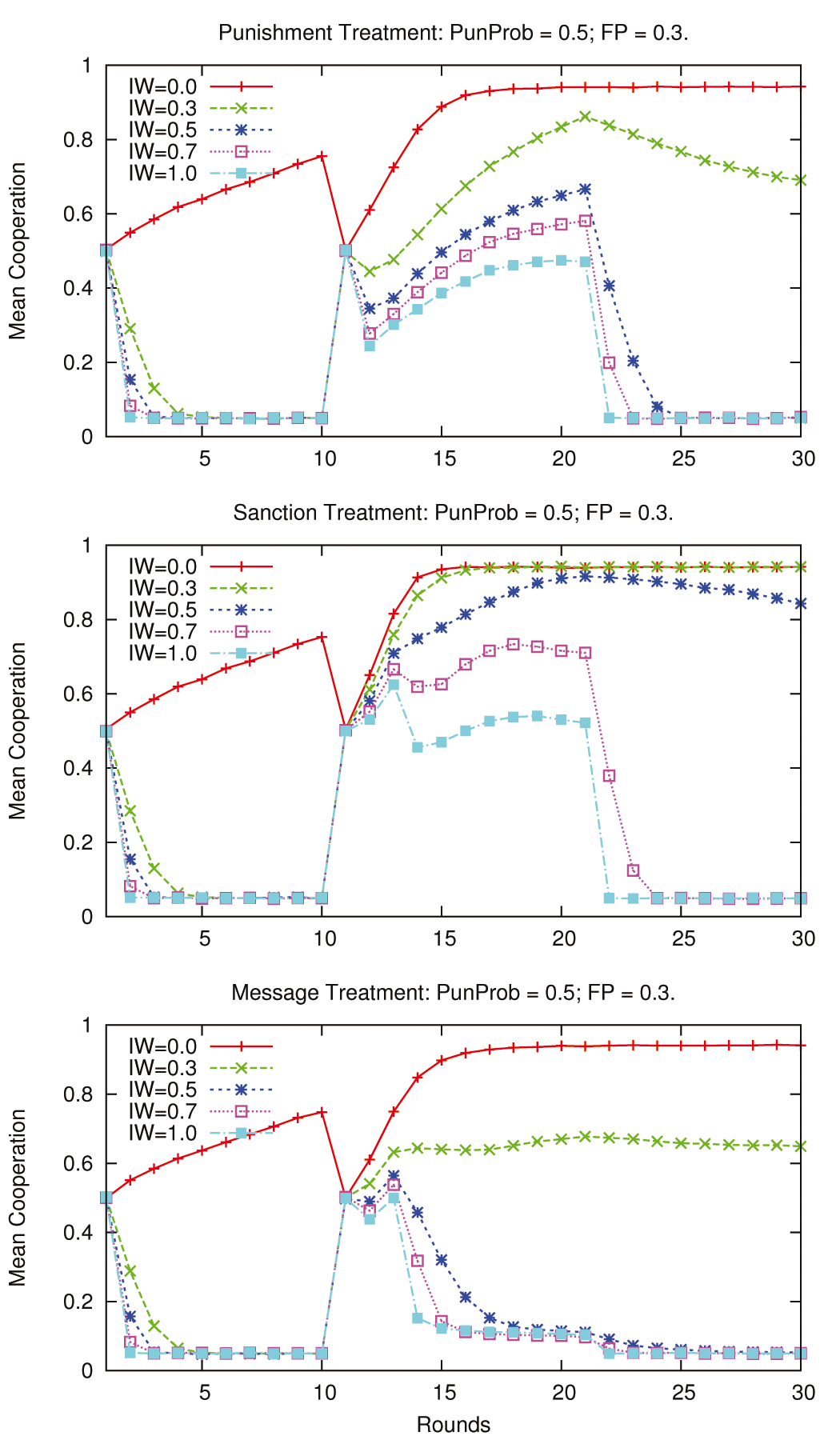

Supplement: Figure S10 — Mean Cooperation along the simulation experiment contrasted with the value of the Individual Weight. (TIF) [file pone.0064941.s010.tif]

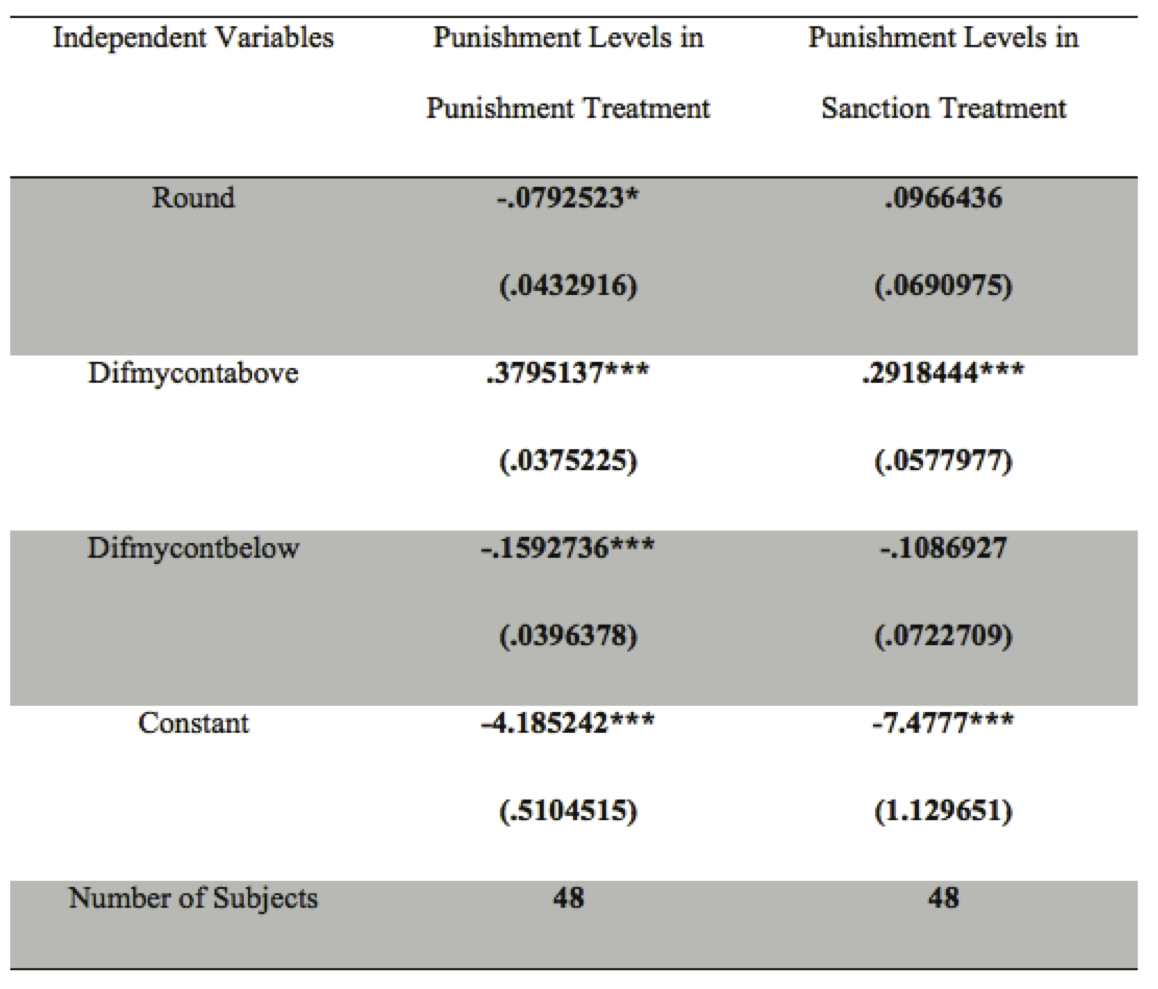

Supplement: Table S1 — Determinants of punishment levels in the punishment and sanction treatments in the Experiments with Human Subjects: random-effects tobit regressions. (TIFF) [file pone.0064941.s011.tiff]

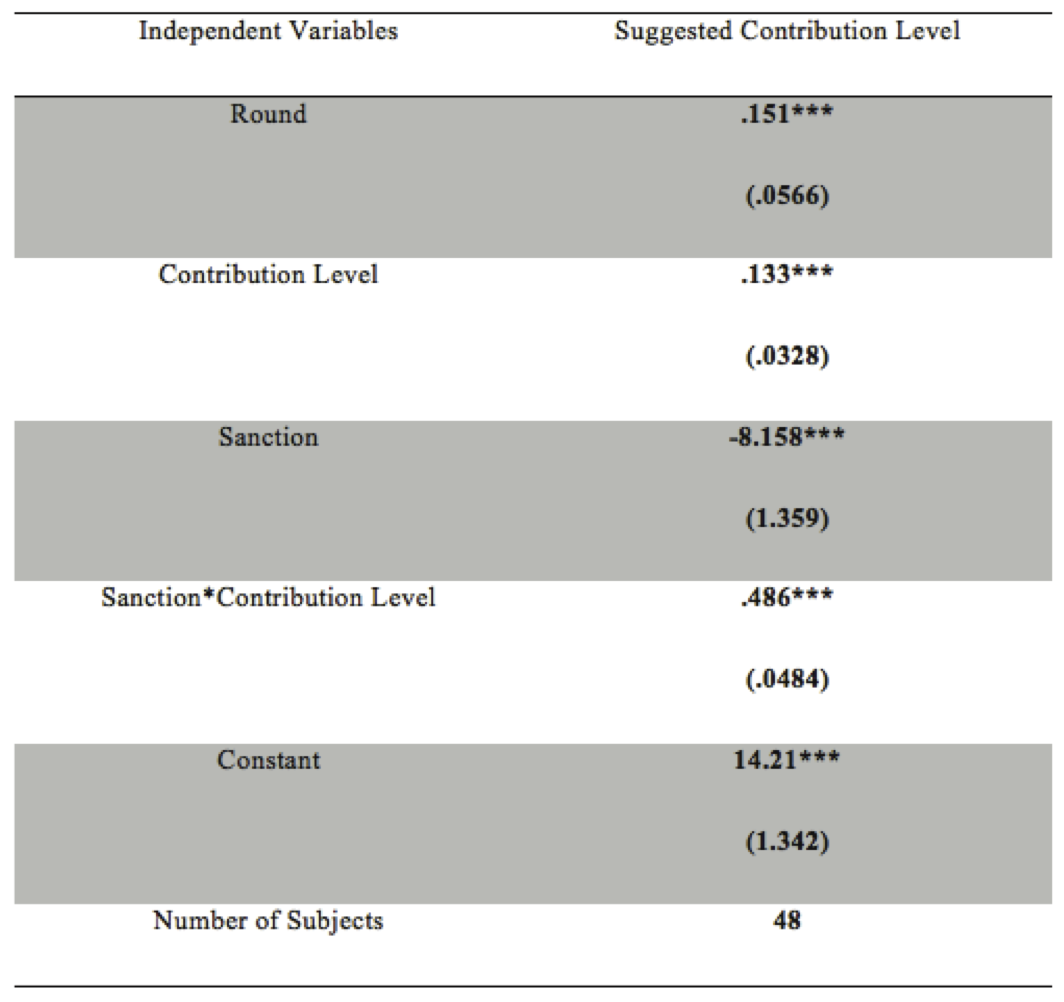

Supplement: Table S2 — Determinants of suggested contributions in the message and sanction treatments in the Experiments with Human Subjects: random-effects tobit regressions. (TIFF) [file pone.0064941.s012.tiff]

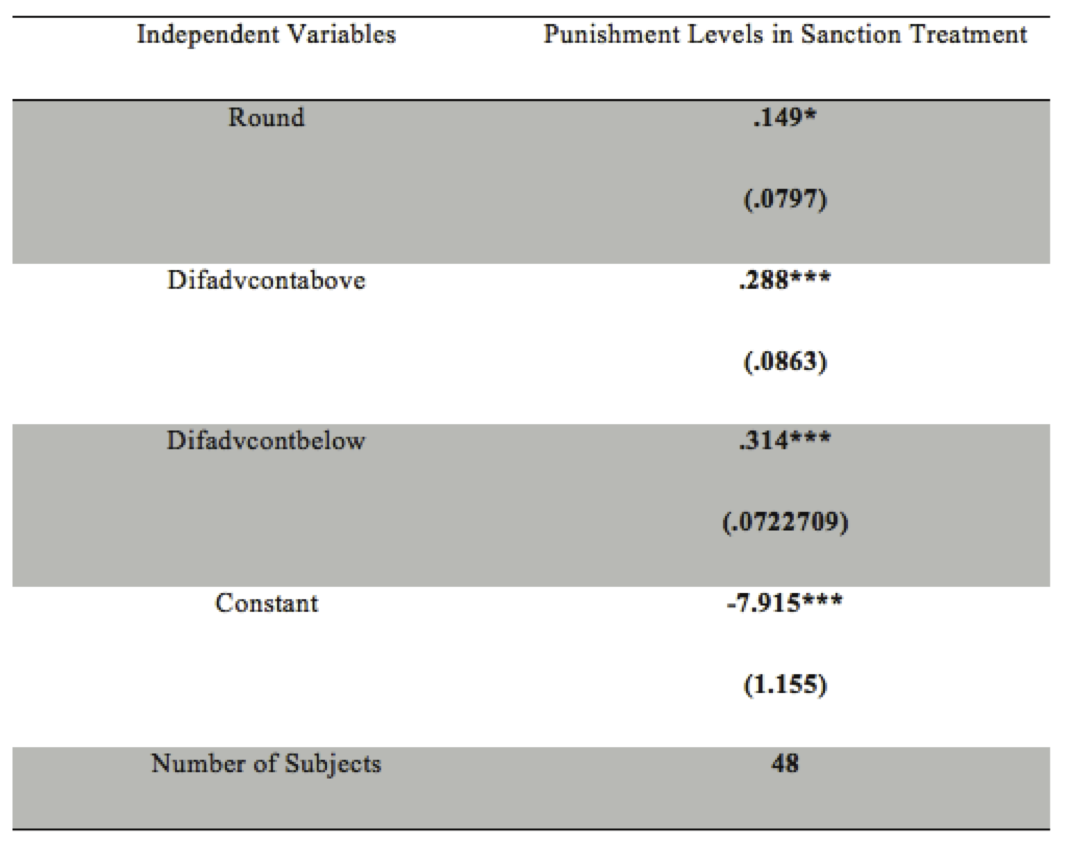

Supplement: Table S3 — Determinants of punishment levels in the sanction treatment in the Experiments with Human Subjects: random-effects tobit regressions. (TIFF) [file pone.0064941.s013.tiff]

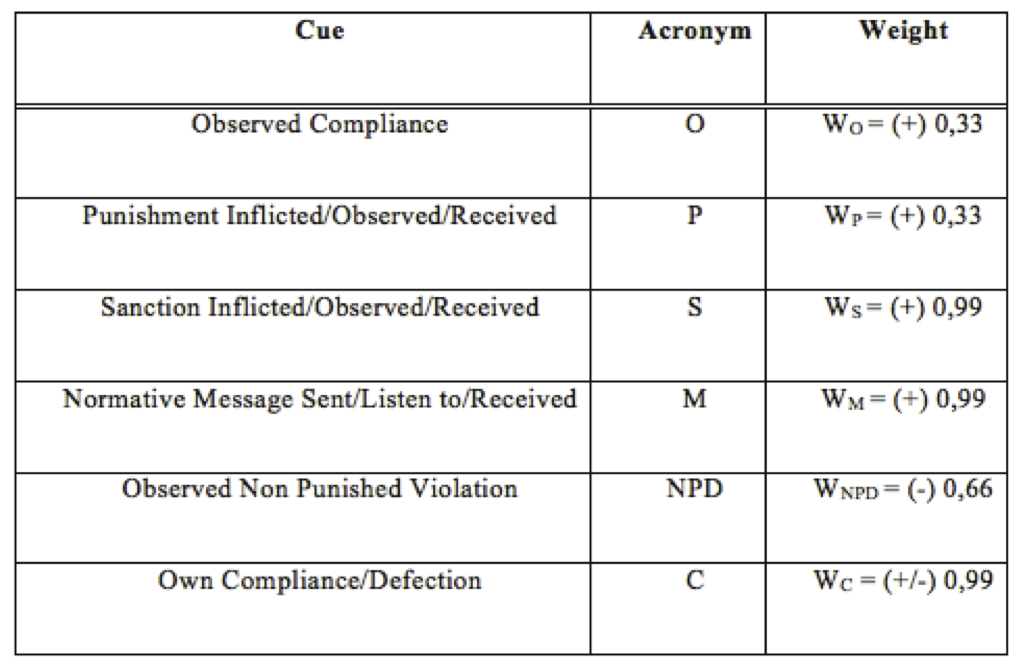

Supplement: Table S4 — Norm Salience Mechanism in the Agent Based Model: Cues and Weights. (TIFF) [file pone.0064941.s014.tiff]
