# Supplementary material for: Punish and Voice: Punishment Enhances Cooperation when Combined with Norm-Signalling
Source: PLoS One. 2013 Jun 12;8(6):e64941. doi: 10.1371/journal.pone.0064941 (PMC3680389; doi:10.1371/journal.pone.0064941)
Supplement: Text S1 — Supporting Information Document. (DOC) [file pone.0064941.s015.doc]

**Supporting Information for Punish and voice: punishment enhances cooperation when combined with norm-signalling**

Giulia Andrighetto^1,2^, Jordi Brandts^3^, Rosaria Conte^1^, Jordi Sabater-Mir^4^, Hector Solaz^5^ and Daniel Villatoro^4^

^1^ Institute of Cognitive Science and Technology, National Research Council (CNR), Rome, Italy

^2^ European University Institute, Florence, Italy

^3^ Universitat Autònoma de Barcelona, Institut d’Anàlisi Econòmica, Consejo Superior de Investigaciones Científicas (CSIC), and Barcelona Graduate School of Economics, Barcelona, Spain

^4^ Institut d'Investigació en Intelligència Artificial, CSIC, Barcelona, Spain

^5^ Nuffield College, University of Oxford, Oxford, United Kingdom

1. **Experiments with Human Subjects**

**Additional analysis of the experimental data**

As shown in Figure 1A of the main paper, in rounds 11-20 contributions are higher in the sanction treatment than in the message treatment[[1]](#footnote-2). Here we give additional information about the frequency with which messages were sent and about the content of the messages that were actually sent in the two treatments.

Figure S1 shows the percentage of subjects that sent a message in the two relevant treatments. One can see that there are no important differences between the two treatments.

The content of messages has two components: the required contribution level “*One should contribute X”* (indicating the demanded token amount between 0 and 20) and a message providing a justification for contributing, which could be one of the following three options: “*because 1) in this way we are all better off; 2) it is what one should do; and 3) if not, it will have consequences for you.”*As indicated in the main paper, these options capture three different reasons for contributing: 1. achievement of a joint benefit; 2. a sense of duty; 3. a purely individualistic motive.

Figure S2 shows average required contribution level for the message and the sanction treatments, conditional on a message being sent. One can see by inspection that there is no difference between the two treatments concerning the required contribution. It is also striking that required levels do not change over time. Subjects simply asked for the maximum (or a level close to it) contribution throughout, with some deviations.

Figure S3 shows which of the three messages mentioned above were sent in the message and the sanction treatments, respectively. What is common to the data shown in the two treatments is that message 1 is the most frequent one: “*One should contribute X* (indicating the demanded token amount between 0 and 20) *because 1) in this way we are all better off*.” Here we also see differences between the two treatments. In the sanction treatment data, subjects use message 1 consistently 80% of the time (or above) over rounds 11-20. In contrast, in the message treatment the use of message 1 starts at a similar level as for the sanction treatment, but then decreases considerably over time. A possible interpretation is that, since in the message treatment messages are less effective in inducing cooperation, subjects switch to the other messages to explore whether they work better.

We now move to show some additional information about punishment behavior in the punishment and sanction treatments. Figure 1B, in the main text, shows that the intensity of punishment inflicted is higher in the punishment than in the sanction treatment. Figure S4 shows which kind of behavior is punished in the two treatments together with the percentages of the different cases.

First, one can see that in the sanction treatment punishment levels are so low that one cannot really refer to any particular pattern. For the punishment treatment, we see that downward deviations are punished more harshly than upward deviations, i.e. punishment is overall of the social type. Observe also that there is somewhat more of a dispersion of deviation levels in the punishment treatment.

**Statistical analysis of the experimental data**

In this section, we provide some statistical description of three important aspects of the use of punishment and suggested contribution levels in our experiments with humans: the determinants of punishment levels in the two relevant treatments (i.e., punishment and sanction), the determinants of suggested contribution levels in the two relevant treatments and the interaction of punishment and suggestions in the sanction treatment.

Table S1 shows the results of our regression study of the determinants of punishment levels. The results can be seen as a complement to the information shown in Figure S4. The variable “Difmycontabove” measures the deviation of the contribution of the punisher with respect to that of the punished when the punisher‘s contribution is above that of the punished and “Difmycontbelow” measures the same deviation for the case where the punisher‘s contribution is below that of the punished.

For the punishment treatment, we see a weakly significant effect of the Round variable, while for the sanction treatment it has no effect. In the punishment treatment, a punisher whose contribution is above that of the punished punishes significantly more the higher the difference. Similarly, a punisher whose contribution is below that of the punished punishes significantly more the higher the difference, but the magnitude of the coefficient of “Difmycontbelow” is only half that of “Difmycontabove,” consistent with the fact that the blue bars on the left side of Figure S4 are much larger than those on the right side of the figure.

The regression results for the sanction treatment show that now only Difmycontabove has a significant effect. “Anti-social punishment” is very weak in this case.

Table S2 shows how a person’s suggested contribution level for the next round depends on the same person contribution level in the current round. It can be seen that the contribution level has a significantly positive effect on the suggestion, controlling for a number of relevant variables.

One can conclude that those participants that ask for high contributions are those who contribute at high levels.

The results shown in Table S3 give insights into how the joint use of messages and punishment interacts with others’ behavior. The independent variable “Difadvcontabove” is between what another group member has contributed in the previous round and what the person who is punishing is suggesting for the current round, for the case where the other contributed more than the punishing person is suggesting. The variable “Difadvcontbelow” denotes the same difference, but for the case where the other contributed less than the punishing person is suggesting. Both variables have a significant effect on the punishment level. The effect of the second variable is quantitatively higher and more natural: the higher the suggestion I am sending you is above what you did in the previous round, the higher the punishment level I apply to you. The significant positive effect of the second variable -“Difadvcontbelow”- can correspond to an attempt of keeping others on their toes.

In conclusion, we find support for the idea that those players who contribute less than asked to are strongly punished.

**2. Agent Based Simulations**

Agent Based Modeling is a relatively young modeling technique that consists in describing a system from the perspective of its constituent units [1-2]. These units (the agents) are modeled as autonomous decision-making entities that interact among them in the context of a computational simulation. The analysis of repeated interaction allows the exploration of emergent dynamics that otherwise would be out of the reach of pure mathematical methods. Agent Based Modeling is especially useful when the behavior of the entities is complex and changes in response to the local environment, exactly the kind of scenario we have in the study of punishment. Agents in the punishment simulation experiment have to make decisions that will influence the behavior of the other participants in the future. In addition, in our model agents’ behavior is also influenced by norm salience, a measure that indicates to each single agent how prominent a norm is within the group. The norm salience value is updated over time by each agent according to both the personal decisions taken by the agents and the normative and social information gathered by observing and communicating with others. This interdependency in the behavior of participants is what makes an analytical approach unfeasible (or at least, extremely complex) for modeling the dynamics of the present simulation experiment and at the same time Agent Based Modeling an ideal tool.

**Agent Based Model description**

Our agent-based simulations are focused on examining the effect of punishment in sustaining cooperation in a social dilemma situation when norm-based communication about what ought to be contributed can be used jointly with the imposition of a material cost.

Agents in the simulation play a variation of the public goods game played by humans presented above. In the simulation the decisions associated with the first and second stages of the game (i.e., how much to contribute, from 0-20; how much to punish, from 0-10) have been simplified. The first choice has been transformed into a binary decision (i.e., to contribute or not); while at the second stage, agents have to decide whether to punish or not and its severity can be high or low.

The main motivation for this adaptation is due to the scope of this research, which consists in understanding the effects of different types of punishment on cooperation rates. As discussed above, we focus on how the different decisions made by subjects affect their normative reasoning. However, a continuum of decisions would introduce another level of complexity for the simulated agents that is out of the scope of this paper, since it would require the semantification of each type of cooperative act (e.g., what level of cooperation should be considered high? Why should different agents understand similarly any particular cooperation levels?). Given the main objective of our research is to observe the effects of different punishment mechanisms in promoting cooperation, we assume that agents only have to choose whether or not to cooperate. With this approach, agents’ decisions are perfectly understandable by the other agents.

At the beginning of the simulation, 48 agents are randomly distributed in groups of four players, which remain unaltered for the rest of the game. A game consists of 30 rounds, divided in three blocks of ten rounds. The first (1-10 rounds) and the third (21-30 rounds) blocks of rounds of the game consist in a standard public goods game in which agents simultaneously have to make the choice to contribute (or not) their endowment of 20 units to the public good. Cooperation choices are binary: each agent chooses whether to cooperate (C) by contributing the whole endowment or to defect by contributing nothing (D). Once all the agents have made their choices, the decisions of the other members of the group are made public. The payoff, P, of each agent, i, of the group is given (as in the case of the game played by humans) by:

$$P_{i}=20-C_{i}+0.4(C_{1}+C_{2}+C_{3}+C_{4})$$

Where C_i_ represents the integer contribution of agent *i* that can only take the value 0 or 20.

In rounds 11-20, treatments differ. Each round consists of two stages. Agents decide to contribute or not all their endowment to the public good. After being informed about the contribution of the other three group members, agents are given the option to react against those who did not contribute. As in the experiments with humans, there are three separate treatments:

1. In the **punishment treatment** each agent, after each round, can assign punishment points to the agents who defected. In our model antisocial punishment is excluded a priori. We are aware that this restriction can affect the outcomes and in future study we intend to consider revenge and antisocial punishment (3). Punishment is costly both for the agent who inflicts punishment and for the one receiving it and its severity can be high or low. High punishment costs 30 units to the punished agent and 10 to the punishing agent; while low punishment costs 5 units to the punished agents and 1,66 to the punishing one. The cost for the punisher is proportional to the damage for the punished and the ratio (1:3) is the same as the one used with the human subjects.
2. In the **message treatment** each agent can, after each round, send to the other group members who defected a message indicating that the norm prescribes to cooperate. Unlike punishment, this message does not affect the earnings of agents sending or receiving it. It is important to notice that in the experiment with humans, to motivate cooperative conduct humans have three different text messages to choose from. However, the message “in this way we are all better off,” which calls for the achievement of a joint-benefit, was by far the most frequently used one. Given that, we decided not to model this additional message in the simulation and assume that the message the agents send to each other is understood without potential misunderstanding. In other words, because the content of the message is supposed to be always the same, what is relevant is if the message is being sent or not.
3. In the **sanctioning treatment**, agents can combine punishment with the normative message.

Since, agents act according to mixed strategies at the end of each time-step the strategies’ probabilities of cooperating, punishing and sending the message are updated. This updating is done on the basis of the payoffs agents obtained in that round and of the social and normative information gathered while interacting with the other agents. In the section *Strategies Updating*, we provide a description of how agents’ decision making works and is updated.

**Norm salience updating**

Agents are endowed with a mechanism allowing them to track how salient a certain norm is. In the present game, the only norm is the norm of cooperation. The salience of the norm is a measure that indicates to the agents how much a norm is prominent within the group, in other words, how important the observance of that norm is considered by members of that group [4-7]. Norm salience has a direct influence on the motivation of the individual to observe the norm.

In our model, norm salience is a parameter endogenously and dynamically updated at every round by each agent according to both the personal decisions taken by the agents and the normative and social information gathered by observing and communicating with others.

The cues that influence the norm salience updating are summarized in Table S4 (“Cue” column). At the end of each interaction agents count the occurrences of each of the cues within their group and aggregate them with different weights (see the “Weight” column in Table S4).

Behavioural or communicative acts that are interpreted as either compliant with or transmitting and enforcing the norm increase the salience of the norm. Those acts that explicitly mention the norm, such as normative messages or sanctions, have a stronger impact on norm salience (i.e., have a stronger weight) than acts in which the normative request is not as explicit, such as punishment. Conversely, unpunished violations reduce the norm salience, by signalling that the group is losing interest in the norm and does not invest in its enforcement anymore. Finally, the acts performed by the individual have a self-enforcing effect on the norm salience.

The social cues contributing to the norm salience updating have been based on the work done by Cialdini et al. [7]. In this work, the authors show the relative effect of different environmental cues (such as, explicit *vs* less explicit normative messages) and conducts (such as, compliant acts, violations, acts of disapproval) in focusing subjects’ attention on the “against-littering” norm and the consequent effects in motivating subjects’ compliance with that norm.

More formally, the function for calculating norm’s salience is the following:

$$Sal^{t}=Sal^{t-1}+\frac{1}{\alpha+\omega}(W_{C}+W_{O}\cdot O+W_{NPD}\cdot NPD+W_{P}\cdot P+W_{S}\cdot S+W_{M}\cdot M)$$

where *Sal^t^* represents the salience of the norm at time t, the number of other agents in the group that the agent has, the normalization value, W_X_ the weights specified in Table 4, and finally O, NPD, P, S, M indicate the registered occurrences of each cue. Salience is in the range of [0,1], 0 representing minimum salience and 1 maximum.

As mentioned above, since every agent has access only to a limited amount of information, the resulting salience value is subjective for each agent, thus providing heterogeneity across agents. This norm salience mechanism enables the agents to monitor and track the strength of the norms in their social environment and to dynamically adapt to them. For example, in an unstable social environment, if a norm decays, our agents are able to detect this change, ceasing to comply with it and adapting to the new state of affairs. Moreover, if norm enforcement suddenly decreases, agents are less inclined to violate those norms that they perceive as highly salient. Perceiving a norm as highly salient is a reason for an agent to continue complying with it even in the absence of punishment. This guarantees a sort of temporary *inertia*, making agents less prone to change their strategy in a pure reactive way.

**Agents’ Strategies Updating**

Previous simulation models have based their functioning on evolutionary algorithms that favor over generations the most fitted agents behavior. Differently to these simulation models where the agents’ adaptation is inter-generational, the implementation of agents whose behavior adapts to the instantaneous actions of their peers is one of the most important contributions of this simulation model. In order for agents to be affected by the behavior of their peers, we need to build the adaptive decision making process that drives agents’ choices.

Depending on the treatment, agents can decide whether or not to cooperate, to punish, and to send messages. These strategies are updated as follows:

**Cooperation probability**

The decision to cooperate or not depends on a probability that varies at each round as a function of the force of both the individual drive and the normative drive.

The **individual drive** approximates instrumental decision-making processes and motivates agents to maximize their own utility, independent of what the norm prescribes, and is updated according to a winner-stay-losers-change algorithm. The more an action increases the agent’s payoffs, the higher the probability it will be chosen. The individual drive directs the choice toward cooperation (C) only when the benefit of defecting is lower than the benefit of cooperating. Agents’ payoffs depend on their actions, and they are lowered according to the costs sustained when imposing punishment or sanction and when receiving them. Agents are given a memory to keep track of the punishments received and, at each time-step, when deciding whether to cooperate or not, they calculate a probability of being punished by checking this memory. If agents anticipate that they can be punished (by finding in their memory that a previous defection was punished), they include the cost of a high punishment into the calculations (as it can be the most extreme value affecting their calculations).

Agents’ decisions are therefore affected directly by the memory they have and how frequently they forget (the Forgetting Probability is an input parameter for our simulation). If they forget at every round, they will never take into consideration the cost of punishment and therefore they will move inevitably towards defection. On the other hand, if they remember everything, after one punishment received they will always take it into consideration, and they will move always towards cooperation. In the message and sanction treatment, the normative message by itself has to be considered as a real menace initially, but it will lose its credibility after two rounds, unless it is supported by the existence of a costly punishment. We can therefore see how the Forgetting Probability also affects this Drive. The algorithm used to update the Individual Drive is the following:

if(defected){

potential_reward_for_cooperation = ((coop+1.0)*8)

potential_reward_for_defection = ((coop*8)+20.0)

}

if(cooperated){

potential_reward_for_cooperation = (coop*8)

potential_reward_for_defection = (((coop-1.0)*8)+20.0)

}

if(remeberMonetaryPunishment || cheapTalk < 2){

potential_reward_for_defection -= High Punishment Cost

}

if(potential_reward_for_defection > potential_reward_for_cooperation){

ID = -1.0

} else {

ID = 1.0

}

Where *defected* means that the agent decided to defect that round, and *cooperated* means that the agent decided to cooperate; *potential_reward_for_cooperation* is the reward that the agent would have gained if it had cooperated, and *potential_reward_for_defection* is the reward that the agent would have gained if it had defected. Both of them are calculated using the number of cooperators represented by coop and the formula presented previously; *remembersMonetaryPunishment* is a flag that is active if the agent previously defected and received material punishment after that defection; *cheapTalk* is an internal variable of each agent that serves to count how many times an agent heard the normative message without observing the corresponding monetary punishment.

Therefore, the Individual Drive algorithm can be summarized as follows: on the basis of the action taken, the agent calculates the potential payoffs he would have received if he had taken the other action (considering the cost of punishment if it applies in the case of defection) and then the individual drive moves towards the action that returns the higher payoff.

The **normative drive** is updated on the basis of the norm salience. The more salient a norm is perceived to be, the higher its strength on the normative drive. In formal terms, the updating of the normative drive is given by:

$$ND=Sal^{t}$$

In our model, agents’ preferences to comply with the norm are conditional on the norm salience. The higher the number of agents complying with the norm and enforcing it, the more salient the norm is perceived to be. Norms, depending on their perceived salience, affect the decision making of the agent and elicit the compliant conduct accordingly.

The cooperation probability changes over time depending on the values that the individual drive and the normative drive take. Moreover, because of the subjectivity of the salience value, this probability varies also across agents, thus generating heterogeneity in their motivation toward cooperation. The tendency to cooperate is always positively affected by the normative drive and possibly by the individual drive if cooperation returns higher payoffs than defection. In this case the two drives complement each other. Conversely, it will be negatively affected by the individual drive when defection returns higher payoffs than cooperation. In this second case, one drive goes against the other.

The resulting probability to cooperate in a specific time *t* is calculated using the following formulas:

$$\begin{matrix} p^{t}=p_{Cooperation}^{t-1}+(ID\cdot IW+ND\cdot NW) \\ \\ p_{Cooperation}^{t}=\left\{\begin{matrix} 0 \\ p^{t} \\ 1 \end{matrix}\begin{matrix} if \\ if \\ if \end{matrix}\begin{matrix} p^{t}\leq0 \\ 0<p^{t}<1 \\ p^{t}\geq1 \end{matrix}\right. \end{matrix}$$

where *p^t^_Cooperation_* is the probability of cooperating at time *t* and, ID and ND are the individual and the normative drive respectively (whose values are adapted at each round), and the IW and the NW are the Individual Weight and Normative Weight. IW and NW are fixed at 0.5 for all the simulations, and represent the importance that each agent assigns to the Individual and Normative Drive. By varying these values different types of agent can be designed, varying from the more individualistic to the more normative types of agents.

Agents follow the algorithm to update their cooperation probability in 95% of the cases. They have a 5% probability to take a random action that represents a mutation in their strategy.

**Punishment and message probability**

Those agents who cooperated during the first stage of the game can decide to punish or send a normative message (or both) to defectors. The probability to punish a defector is inversely proportional to the number of defectors [8] within the group. The higher the number of defectors, the lower the probability to punish.

We extracted the agents’ initial probability of sending monetary punishments (equals to 50%) from the results obtained by humans in the very first round of the experiment. These results are not affected by any parameter of the experiment. Once an agent decides to punish the defector, it has to decide how severely to punish. We have categorized the severity of punishment in three levels: high, low, and no punishment. Attending also to empirical results obtained with humans, agents will initially send high punishments in the Punishment treatment and low punishments in the Sanction Treatment. Then, they will change the severity according to its effectiveness: if the number of defectors decreases, agents will reduce the severity of punishment accordingly; Conversely, if the number of defectors increases, so will the punishment severity.

Once the decision about the level of punishment has been inferred, the probability to send the normative message is proportional to the norm salience. The more the norm is salient, the higher the probability to send the normative message to those who do not respect it.

**Extended technical description of the agent-based simulations**

All the results presented in this section are calculated averaging the results of 1000 independent simulations with the same set of parameters defined in each treatment.

**Dynamics of the individual and normative drives**

Figure S5 shows how the individual and the normative drives vary along the rounds in the simulation for the three different treatments. The individual drive ranges from [-1,1], as it can reduce or increase the tendency to cooperate; on the other hand, the normative drive ranges from [0,1], affecting only positively the tendency to cooperate of the agents. The values presented in the figure are the mean of the agents’ individual and normative drives at each round.

During the first ten rounds, when no punishment is allowed, the individual drive takes its most strength negative value, decreasing the cooperation probability. From round 11 to 20, once agents, depending on the specific treatment, can use punishment, sanction or messages for reacting against defectors, different effects on the individual and normative drives can be observed.

In the punishment treatment, the normative drive slowly increases because of the different normative cues (such as cooperative acts and punished defectors) observed affecting the norm salience (see Table S4). The individual drive also takes a positive value because punishment makes cooperation the most beneficial act.

In the sanction treatment, the normative drive increases much faster than in the previous treatment, because of the explicit normative message strongly affecting the norm salience (see Table S4). Initially, the individual drive has a positive value, however, once agents start to cooperate consistently, they stop punishing (because of the lack of free-riders). This lack of punishment affects the benefit-cost calculation performed when updating the Individual Drive. Defection turns to be the more advantageous action. As a consequence, the individual drive takes a negative value that reduces the probability of cooperation. As agents are endowed with the same weight for both drives, the Normative drive prevails over the Individual drive in these situations, resulting yet in an increase of the cooperation probability.

In the message treatment, we can observe the “menacing” aspect of the normative message. As explained previously, the normative message is considered by the agents as a real menace initially (i.e., it counts as a monetary punishment received in the benefit-cost calculation), resulting in a positive value of the Individual Drive (as cooperating is the most beneficial action). However, when the menacing aspect disappears, the Individual Drive takes a strong negative value, which overcomes the Normative Drive, causing a decrease of the cooperation probability.

**Agents’ reactions in the second phase: amount of punishments, sanctions and messages sent**

Figure S6 shows the amount of monetary punishments - high and low - and normative messages in each of the three treatments. Even though the number of high punishments is barely distinguishable in the punishment and sanction treatments, we can observe a significant difference in the low punishments, which is notably lower in the sanction treatment. Moreover, we observe that the number of normative messages (number of reactions in *Sanction M* and *Message M*) sent in the sanction treatment is higher than in the message treatment after round 14, when the cooperation collapses in the message treatment.

**Average payoff per agent per treatment**

In Figure S7 the average payoff per agent per treatment is shown. Average payoffs in the sanction treatment are higher than in the other two treatments and higher than when punishment is not available.

In the punishment and sanction treatments, agents can adapt the intensity of punishment and this causes different average payoffs (as punishment implies a cost both for the punisher and the punished). We can observe that at the beginning of the second block (i.e., in round 11) the average payoffs are higher in the message treatment, as sending the normative message does not imply any costs, but it has only a temporary positive effect on cooperation. However after four rounds, as agents start defecting, the average payoffs in the message treatment decrease and become lower than those in the sanction treatment. Agents in the sanction treatment apply very few punishments and even when applied they are very low, resulting in a significant saving of resources and in an increase of the average payoff.

Finally, the average payoffs in the punishment treatment are very low as agents’ probability to defect is higher than in the other two treatments and consequently punishers spend significant amount of resources trying to deter defectors.

**Exploring the Parameter Space**

Our simulation model needs to be fed with 3 different parameters. These parameters are:

- Individual Weight (IW) $\in$ [0,1]
- Initial Monetary Punishment Probability (PP) $\in$ [0,1]
- Forgetting Probability (FP) $\in$ [0,1]

Different configurations of these values will have different impacts on the overall analyzed behavior. We have made a parameter space study and in this section we present the resulting effects.

In all the following experiments, the simulation is composed of 48 subjects, which are randomly divided in groups of 4 and remain constant.

The first simulation experiment concerns the initial monetary punishment probability (PP). In order to observe its effects, we fix the value of the other parameters and vary only this one, in all three treatments. The Individual Weight (IW) is fixed to 0.5, and consequently the Normative Weight (NW) is also fixed to 0.5. Finally, the Forgetting Probability (FP) is fixed to 0.3. The effects of this parameter can be observed starting from round 11, when agents are allowed to punish. As shown in Figure S8, the message treatment is not affected by the variation of this value because of the impossibility to inflict punishment in such treatment. However, we observe an effect on the other two treatments. In both the punishment and the sanction treatment, the cooperation rates turn out to be proportional to the initial punishment probability. Higher initial probabilities of punishment will affect agents’ decision making by altering their individual and normative drive. However, the effect of PP in promoting cooperation is stronger in the sanction than in the punishment treatment. This is because monetary punishment is combined with messages that have an effect in increasing the norm salience and consequently the normative drive.

The second simulation experiment allows us to analyze the effect of the Forgetting Probability (FP). By fixing the Individual Weight (IW) to 0.5, and the Initial Monetary Punishment Probability (PP) to 0.5, we vary the forgetting probability. As in the previous experiment, the effects of the forgetting probability parameter are only noticeable after round 10. All three treatments show different dynamics with respect to the variation of this parameter. As shown in Figure S9, the cooperation rates in the punishment and the sanction treatments are inversely proportional to the value of the parameter. The more frequently agents forget, they will be more frequently they are tempted to defect, as they will not take into account punishment in their cost and benefit calculation. For this reason with higher forgetting probabilities the average cooperation rates are lower, as agents defect more frequently. In the message treatment however, the effect is different. The reason is because of the “cheap talk” effect. When agents receive a normative message, without a monetary punishment associated, they consider this message as a reliable prescription, but only for 2 rounds. While the message is trusted, agents incorporate the cost associated to punishment into their calculations, and decide accordingly. If after these two rounds agents do not observe any defection being punished, they consider the message as “cheap-talk” and they do not include punishment in their cost and benefit calculation anymore. However, the more they forget, the more frequently they will consider the message as reliable.

The third simulation experiment fixes the PP to 0.3 and the FP to 0.5, to analyze the effects of the individual weight (and the consequent normative weight). Figure S10 shows that cooperation rates are inversely proportional to the individual weight. The explanation is due to the own nature of the individual weight, as it provides a higher importance to the payoffs and material benefits obtained, reducing the importance of the normative weight, that naturally influences individuals towards cooperation. In the message treatment however, we can observe that whenever the individual weight is stronger than the normative weight, cooperation does not emerge. Conversely, when the normative weight is stronger, the normative messages have enough effect on the decision making for cooperation to emerge.

**References**

1. Bonabeau B (2002) Agent-Based Modeling: Methods and Techniques for Simulating Human Systems. PNAS 99:7280-7287.

2. Axelrod R (1997) The Complexity of Cooperation: Agent-Based Models of Competition and Collaboration. Princeton, NJ: Princeton Univ. Press.

3. Rand D.G., Armao JJ 4th, Nakamaru M, Ohtsuki H (2010) Anti-Social Punishment can Prevent the Co-Evolution of Punishment and Cooperation. Journal of Theoretical Biology 265: 624-32.

4. Andrighetto G, Villatoro D (2011) Beyond the Carrot and Stick Approach to Enforcement: An Agent-Based Model. In B. Kokinov B, Karmiloff-Smith A, Nersessian N J, editors European Perspectives on Cognitive Science. Sofia: New Bulgarian University Press.

5. Bicchieri C (2006) The Grammar of Society: The Nature and Dynamics of Social Norms. Cambridge: Cambridge University Press.

6. Xiao E, Houser D (2011) Punish in Public. Journal of Public Economics 95, 1006-1017.

7. Cialdini R.B., Kallgren C.A., Reno R.R. (1990) A Focus Theory of Normative Conduct: a Theoretical Refinement and Reevaluation of the Role of Norms in Human Behavior. Advances in Experimental Social Psychology 24: 201–234.

8. Rosas A (2008) The Return of Reciprocity: a Psychological Approach to the Evolution of Cooperation. Biology and Philosophy, 23: 555–566.

**Supporting Information Legends**

**Text S1: Supporting Information Document**

**Figure S1. Percentage of individuals that sent a message over rounds 11-20 in the Experiments with Human Subjects.**

**Figure S2. Average required contribution in tokens over rounds 11-20 in the Experiments with Human Subjects.**

**Figure S3. Percentages of the three verbal messages sent in the message and sanction treatment over rounds 11-20 in the Experiments with Human Subjects.**

**Figure S4. Punishment intensity in the Experiments with Human Subjects depending on punished subject’s contribution minus that of punisher.**

**Figure S5. Dynamics of the Individual and Normative Drives in the Agent Based Model.**

**Figure S6. Amount of punishments, sanctions and messages sent in the Agent Based simulation.**

**Figure S7. Average Payoffs along the Agent Based simulation.**

**Fig. S8. Mean Cooperation along the simulation experiment contrasted with the value of the Initial Punishment Probability.**

**Fig. S9. Mean Cooperation along the simulation experiment contrasted with the value of the Forgetting Probability.**

**Fig. S10. Mean Cooperation along the simulation experiment contrasted with the value of the Individual Weight.**

**Table S1. Determinants of punishment levels in the punishment and sanction treatments in the Experiments with Human Subjects: random-effects tobit regressions.**

**Table S2. Determinants of suggested contributions in the message and sanction treatments in the Experiments with Human Subjects: random-effects tobit regressions.**

**Table S3. Determinants of punishment levels in the sanction treatment in the Experiments with Human Subjects: random-effects tobit regressions.**

**Table S4. Norm Salience Mechanism in the Agent Based Model: Cues and Weights.**

1. In round 11 of the laboratory experiment, differences in contribution levels cannot be the result of different punishment levels or normative messages. Nevertheless no-random differences in behavior are possible, since the participants have read the new instructions pertaining to rounds 11-20. The different instructions for the three treatments lay out the different possibilities of punishing and sending messages in the different cases and subjects may be influenced by these differences. In particular, it is possible that subjects in the sanction treatment anticipate that higher contribution levels will be required in this case. However, the results of the three pair-wise Mann-Whitney test finds no difference between contribution levels in the three treatments in round 11 (p=0.3644 for message vs. sanction, p=0.1204 for punishment vs. sanction, p=0.6102 for message vs. punishment). [↑](#footnote-ref-2)
